# Single-Switching Reachable Operation Points in a DC-DC Buck Converter: An Approximation from Time Optimal Control

**DOI:** 10.3390/mi11090834

**Published:** 2020-08-31

**Authors:** Ilya Dikariev, Fabiola Angulo, David Angulo-Garcia

**Affiliations:** 1 Department of Optimization, Campus Cottbus, Institute of Mathematics, Brandenburg University of Technology, Platz der Deutschen Einheit 1, 03046 Cottbus, Germany; 2 Departamento de Ingeniería Eléctrica, Electrónica y Computación—Bloque Q, Campus La Nubia, Facultad de Ingeniería y Arquitectura, Universidad Nacional de Colombia—Sede Manizales, Manizales 170003, Colombia; fangulog@unal.edu.co; 3 Grupo de Modelado Computacional—Dinámica y Complejidad de Sistemas, Instituto de Matemáticas Aplicadas, Universidad de Cartagena, Carrera 6 # 36-100, Cartagena de Indias 130001, Colombia; dangulog@unicartagena.edu.co

**Keywords:** DC-DC synchronous buck converter, time optimal control, pontryagin maximum principle, control synthesis

## Abstract

In this paper, we study the time optimal control problem in a DC-DC buck converter in the underdamped oscillatory regime. In particular, we derive analytic expressions for the admissible regions in the state space, satisfying the condition that every point within the region is reachable in optimal time with a single switching action. We then make use of the general result to establish the minimum and maximum variation allowed to the load in two predefined design set-ups that fulfills the time optimal single switching criteria. Finally, we make use of numerical simulations to show the performance of the proposed control under changes in the reference voltage and load resistance.

## 1. Introduction

In power electronics, DC-DC power converters are essential components in both industrial and day-to-day applications, for instance, fuel and photovoltaic cells [1,2], hybrid vehicles [3], mobile devices [4,5], and many others (see [6] for a review on recent applications).

In particular, the step-down or buck converter is of special interest for the power supply of electronic devices. For this reason, a wide variety of controllers have been proposed for this converter [7], which provide suitable solutions for a particular design purpose: for instance, sliding mode control [8] allows for the robust operation of the converter to unmodeled dynamics, PID based controllers in averaged models [9] enables the use of well-established linear feedback theory and controllers designed using contraction theory can guarantee global stability of the converter [10], just to mention few. However, several modern applications of the buck converter have the specific requirement of fast dynamic response, which is not always guaranteed with the designs that are mentioned above. For example, in Dynamic Voltage Scaling (DVS) [11] and RF amplification [12], it is essential that the output of the converter is reached as fast as possible.

The problem of driving the system towards a desired operation point in minimum time is regarded as the Time-optimal control problem. Time optimal problems can be solved using the Pontryagin’s maximum principle [13,14] and this approach has been extensively used in order to minimize transient behavior in power converters, allowing to find optimal switching surfaces in the boost converter [15], the buck-boost [16], and the buck converter itself in some particular conditions of operation [11,17]. An important question in the time-optimal problem is: given an initial condition, is the system able to reach the target state with a single-switching in optimal time (SSOT for short), before shifting to the use of the high-frequency controller to maintain the system in the vicinity of the desired point?. Indeed, a comprehensive analytic study on the conditions under which the buck, boost, and buck-boost topologies are able to evolve their dynamics in a single switch was first investigated in [18]. However, it was only possible to draw conclusions on the number of switches required to reach the desired output for the buck converter in a low resistance (high load) regime where the dynamics of the buck converter behaves as an overdamped system and the Pontryagin’s maximum principle allows for a straightforward analysis.

Nevertheless, any important applications of the buck converter are associated with low output currents where the dynamics of the buck converter may behave as an underdamped oscillator [19]. The voltage specifications of mobile devices working in stand by mode is a prominent example of an application of the buck converter in the light load regime, where the efficiency of the converter is of utter importance for minimizing the battery drain [20,21,22,23].

The problem of constructing a-priori the subset of the state space that guarantees SSOT transitions remains an open problem for the buck power converter in the low load regime. In this paper, we aim to construct such a subset based on the knowledge of the dynamical properties of the system, that can be easily exploited in canonically transformed coordinates. For this purpose, the paper is organized as follows: in Section 2, we present the mathematical preliminaries associated to time-optimal control and the description of the synchronous buck power converter. In Section 3, we formalize the statement of the problem and develop a geometrical framework to find the region of the state space where it is possible to reach output in optimal time with a single switching action. Moreover, in Section 4, we present some practical implementation examples where we make use of the derived expressions for time optimal regions. Additionally, we present numerical simulations showing the performance of the proposed control in Section 5 and discuss some final remarks in Section 6.

## 2. Preliminaries

### 2.1. Buck Power Converter

**Figure 1:**
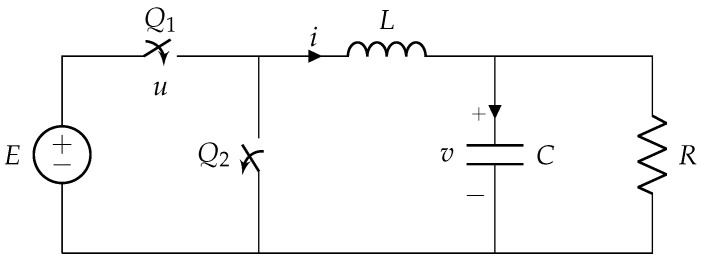
Schematic diagram of a buck power converter.

Figure 1 depicts a simplified version of a synchronous buck power converter. In this system, internal resistances are disregarded and the switches $Q_{1}$ and $Q_{2}$ are coupled in such a way that when $Q_{1}=1$ (ON), $Q_{2}=0$ (OFF) and vice-versa. The equations describing this dynamical system are

(1) $$\left(\begin{array}{c} \dot{v}\\ \dot{i} \end{array}\right)=\left(\begin{array}{cc} -\frac{1}{RC} & \frac{1}{C}\\ -\frac{1}{L} & 0 \end{array}\right)\left(\begin{array}{c} v\\ i \end{array}\right)+\left(\begin{array}{c} 0\\ \frac{E}{L} \end{array}\right)u$$

where *R* is the load resistance, *C* is the capacitor’s capacitance, *L* is the coil’s inductance, and *E* is the voltage provided by the power source. The state variable *v* corresponds to the voltage across the capacitor and *i* quantifies the current flowing through the inductor. The control signal *u* takes values in the discrete set {0,1}. When u=1 the main switch $Q_{1}$ is closed (ON) and the circuit is fed by the input voltage *E*. When u=0 the main switch is open (OFF) and the power source (input voltage) does not feed the system. In this case, the load is being fed by the capacitor and the inductor. For simplicity, we will perform a first transformation that maps the original system (1) into a dimensionless framework by means of the following similarity transformation $x=M^{-1}{\left(v,i\right)}^{T}$ [24], where

(2) $$M=\left(\begin{array}{cc} E & 0\\ 0 & \frac{E}{\sqrt{L/C}} \end{array}\right)$$

Additionally, we perform a normalization in time such that $t=\tau /\sqrt{LC}$—for simplicity in notation we will use *t* as normalized time and τ as the real one-. With these transformations, a new and unique parameter $\gamma =\frac{1}{R}\sqrt{\frac{L}{C}}$ holds the information of the parameters in the system, allowing for us to rewrite the equations as:

(3) $$\left(\begin{array}{c} {\dot{x}}_{1}\\ {\dot{x}}_{2} \end{array}\right)=\left(\begin{array}{cc} -\gamma & 1\\ -1 & 0 \end{array}\right)\left(\begin{array}{c} x_{1}\\ x_{2} \end{array}\right)+\left(\begin{array}{c} 0\\ 1 \end{array}\right)u$$

or in a compact form as $\dot{x}=Ax+Bu$.

This system has two possible equilibria, the first one is given by $\left(x_{1},x_{2}\right)=\left(0,\;\;0\right)$ obtained for u=0 and a second one, given by $\left(x_{1},x_{2}\right)=\left(1,\;\;\gamma \right)$ obtained when u=1. By switching the MOSFETs at a high frequency it is possible to obtain a new equilibrium point corresponding to the averaged value $\left(x_{1}^{*},\;\;x_{2}^{*}\right):=\bar{u}\left(1,\;\;\gamma \right)$, where $\bar{u}$ is the average value of *u*, and turns out to be the normalized reference output $x_{1ref}=V_{ref}/E$. Depending on the parameter values, this equilibrium can be either a focus (complex eigenvalues obtained for 0<γ<2) or a node (real eigenvalues obtained for γ≥2). The quantity γ is closely related with the quality factor of the circuit (*Q*-factor). A value of γ>2 is equivalent to an overdamped circuit with Q<1/2, also at γ=2, Q=1/2 and the circuit is critically damped. Finally, as γ decreases below 2, the quality factor of the circuit increases unbounded and Q→∞ when γ→0.

As previously pointed in the introduction, optimal control has been well studied in the case in which the system behaves as an overdamped circuit (node type) [11]. However, when the equilibrium of the buck converter behaves as a focus it is not possible to generically establish the number of switches that the system will perform before reaching the operation point. In what follows, we present the theoretical basis of time optimal control with the aim of finding the subset of the state space that can be reached in SSOT.

### 2.2. Time Optimal Control

We recall the system (3) and let us assume that the control function u(t) could take values from the convex set U=[0,1]⊂R. The following part is based on Boltyanskiy’s theoretical result for synthesis of optimal control in two-dimensional linear systems [14]. The main result is that the control signal u(t) has to be “bang-bang” in order to be time-optimal. In other words, it has to be switched about the extreme points of the convex polygon *U* of control space. In our case (3), this polygon is one-dimensional and consists of a segment of line with extreme points u(t)=0 and u(t)=1, which precisely correspond to the allowed values of *u* (ON and OFF) of the switches. As we will see later, the time between commutations has to be exactly T/2, i.e., half of the oscillation period *T* of the system (3), except for the very first and the very last switch which could be less than T/2. In our case of study, we analyze time optimal trajectories that consist only of one switching actions, namely u=1→u=0 or u=0→u=1.

We recall the Pontryagin maximum principle to better understand the reason why the control function *u* needs to behave as described above [14]. Let us first define the Hamiltonian of a dynamical system $\dot{x}=f\left(x,u\right)$ as

(4) H(ϕ,x,u)=〈ϕ,f(x,u)〉.

where ϕ is the so-called adjoint function. The goal is to find an optimal control $u^{*}$ which transfers the system from some point $x=x_{0}$ to a point x=0 in a minimal time. According to the Pontryagin’s maximum principle, the optimal solution $\left(x^{*},u^{*}\right)$, together with its corresponding adjoint function $\phi^{*}$ satisfy the following equations:

(5) $$\begin{array}{cc} \dot{x}= & \frac{\partial H}{\partial \phi }\\ \dot{\phi }= & -\frac{\partial H}{\partial x}. \end{array}$$

Applying this result on (3), expressed in the form $\dot{x}=Ax+Bu$, we obtain

(6) $$\begin{array}{cc} \dot{x} & =Ax+Bu\\ \dot{\phi } & =-A^{T}\phi . \end{array}$$

Moreover, the optimal solution $u^{*}$ needs to maximize the Hamiltonian (4) for almost every *t*, namely:

(7) $$\begin{array}{c} H\left(\phi^{*},x^{*},u^{*}\right)=\underset{u\in U}{\sup}H\left(\phi^{*},x^{*},u\right) \end{array}$$

which implies

(8) $$\begin{array}{cc} {\phi^{*}}^{T}\left(Ax^{*}+Bu^{*}\right) & =\underset{u\in U}{\sup}{\phi^{*}}^{T}\left(Ax^{*}+Bu\right) \end{array}$$

(9) $$\begin{array}{cc} {\phi^{*}}^{T}Bu^{*} & =\underset{u\in U}{\sup}{\phi^{*}}^{T}Bu. \end{array}$$

As stated in Section 2.1, we are interested in solutions with γ∈[0,2), i.e., with underdamped oscillatory dynamics. Accordingly, from the Equation (6), we can directly deduce the adjoint solution $\phi^{*}$, as follows:

(10) $$\begin{array}{c} \phi^{*}=re^{\lambda t}\left(\begin{array}{c} \cos(\mu t+\alpha )\\ \sin(\mu t+\alpha ) \end{array}\right) \end{array}$$

with some real α,r. Here, λ is the damping envelope and μ=2π/T, the damped angular frequency of oscillation. This means that the vector $\phi^{*}$ oscillates with the same period *T*, as the characteristic period of the original system (3) around the origin, since the imaginary part of the eigenvalues of $-A^{T}$ and *A* are the same. Now, we take a look to the Equation (9). The goal is to maximize the scalar product ${\phi^{*}}^{T}Bu$. When the vector $\phi^{*}\left(t\right)$ appears in the upper half-plane of $R^{2}$, ${\phi^{*}\left(t\right)}^{T}Bu$ is maximum if u=1. Similarly, when the vector $\phi^{*}\left(t\right)$ belongs to the lower half-plane, the scalar product is maximized with u=0. Unfortunately, it is not possible to explicitly find the initial point $\phi^{*}\left(0\right)$, which makes difficult the calculation of the time in which the control u(t) has to be switched for the first time. What we can assure is that u(t) switches every T/2 except for the very first, and the very last time. To solve this problem, we will make use of the solution proposed by Boltyansky in the same work [14], which consists of constructing the set of all possibles optimal trajectories from the final point x=0 backwards in time.

## 3. Single-Switching in Optimal Time (SSOT) Region in the Buck Converter

In a buck power converter, we are interested in driving the system towards an operation point given by: $\left(x_{1}^{*},\;\;x_{2}^{*}\right):=\left(x_{1ref},\;\;\gamma x_{1ref}\right)$ with $x_{1ref}=V_{ref}/E$. $V_{ref}$ is such that

(11) $$x_{1ref}\in \left[u_{m},u_{M}\right],\;\mathrm{with}\;0\leq u_{m}\leq u_{M}\leq 1.$$

From a practical point of view, $u_{m}=0.1,u_{M}=0.8$, the values that are outside this range are usually not desired. Moreover, we are only interested in values of γ∈(0,2), leading to a focus-type of dynamics. To this end, we shall define the area of the state space that include the set of all possible desired points given the restrictions above as $P_{g}$:

(12) $$P_{g}:=\left\{\left(x_{1},x_{2}\right)\;|\;u_{m}\leq x_{1}\leq u_{M}\cap \;x_{2}>\gamma_{1}x_{1}\cap \;x_{2}<\gamma_{2}x_{1},\;0<\gamma_{1}<\gamma_{2}<2\right\}$$

**Figure 2:**
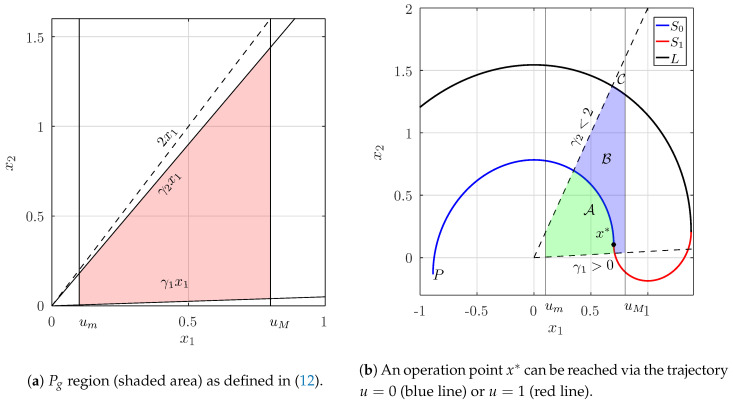
$P_{g}$ region and generic partition of $P_{g}$ in the sets A, B and C.

An schematic illustration of $P_{g}$ is shown in Figure 2a.

We will focus on the case in which the system is operating at a given point and, due to a variation in the system’s parameters, we need to drive the dynamics towards a new operation point in a single switching action. With this, we are effectively disregarding far from equilibrium dynamics and restricting our analysis to the subset $P_{p}\subset P_{g}$ which can be time-optimally driven into $P_{p}$ itself with a single commutation (u=1→u=0 or viceversa). This subset will depend on the changes of γ and $x_{1ref}$.

Let us focus on Figure 2b and the generic operation point denoted by $x^{*}=\left(x_{1}^{*},x_{2}^{*}\right)$. This point can only be reached via a trajectory with u=0 or with u=1. The parts of the trajectories that reach $x^{*}$ in less than T/2 define indeed the optimal switching surface composed by the trajectory segments $S_{0}$ and $S_{1}$.

In order to construct the $P_{p}$ set, let us integrate backwards in time during T/2 starting from $x^{*}$ and u=1. This generates the switching surface $S_{1}$. For the trajectory to be time optimal, the control action cannot be active for more than T/2, therefore, further backward integration has to be made with u=0 leading to the trajectory segment *L*, as stated in Section 2.2. A similar construction can be performed starting with u=0, which generates the switching surface $S_{0}$ by backwards integration during T/2. Nevertheless, it turns out that the furthest point of $S_{0}$, denoted in the scheme as *P* will always lay in the third quadrant for any operation point $x^{*}$ in $P_{g}$. For this reason, the construction described before, divides the set $P_{g}$ in three sets: A,B,C (see Figure 2b). The set C turns out to be empty for some operation points $\left(x_{1}^{*},x_{2}^{*}\right)$, and will be of interest later.

Now, starting from any point of the region A, the point $x^{*}$ can be reached with a control sequence u=1→u=0 (i.e., with a single switching). Every trajectory initiated by u=1 starting at any point in A will intersect the switching surface $S_{0}$ in time t<T/2 and, consequently, reach $x^{*}$ in optimal time $t^{*}<T$. In a similar way, if an initial point lays within the set B then the optimal control is the sequence u=0→u=1.

The last set C contains the points that require *two* switches to reach $x^{*}$ in optimal time in the following way:

(13) $$u\left(t\right)=\left\{\begin{array}{cc} 0, & t\in [0,t_{0})\\ 1, & t\in [t_{0},t_{0}+T/2)\\ 0, & t\in [t_{0}+T/2,t_{0}+T/2+t_{1}] \end{array}\right.$$

with some positive $t_{0},t_{1}$. To any operation point *x* we define the corresponding set $C_{x}$ as depicted in the Figure (note, that the set $C_{x}$ could be empty for some *x*). Our task now is to calculate the union

(14) $$\begin{array}{c} C_{0}=\underset{x\in P_{g}}{⋃}C_{x}, \end{array}$$

which define the inadmissible set. The *admissible* set $P_{p}$ will then be $P_{g}\setminus C_{0}$.

With the aim of finding $C_{0}$, we will apply the following canonical transformation to the system (3) with a properly calculated matrix *P*:

(15) $$\begin{array}{c} y=P^{-1}x,\;P=\left(\begin{array}{cc} \frac{\gamma }{2} & \frac{\sqrt{4-\gamma^{2}}}{2}\\ 1 & 0 \end{array}\right), \end{array}$$

in such a way to represent the system in the canonical form

(16) $$\begin{array}{c} \dot{y}=\left(\begin{array}{cc} \lambda & -\mu \\ \mu & \lambda \end{array}\right)y+P^{-1}\left(\begin{array}{c} 0\\ 1 \end{array}\right)u, \end{array}$$

where $\lambda =-\frac{\gamma }{2}$ and $\mu =\frac{1}{2}\sqrt{4-\gamma^{2}}$. With this transformation, it is easy to see that, for u=0 (corresponding to the case in which equilibrium is at the origin), the radial distance of the points along any trajectory of the system can be expressed in polar coordinates as

(17) $$\begin{array}{c} r=r_{0}e^{\left(\alpha +\alpha_{0}\right)\frac{\lambda }{\mu }}. \end{array}$$

Similarly, when u=1, the equilibrium is now translated to the point $F=-P^{-1}A^{-1}{\left(0,1\right)}^{T}$ and radius of each point in the trajectory is also given by (17) measured from *F*.

**Figure 3:**
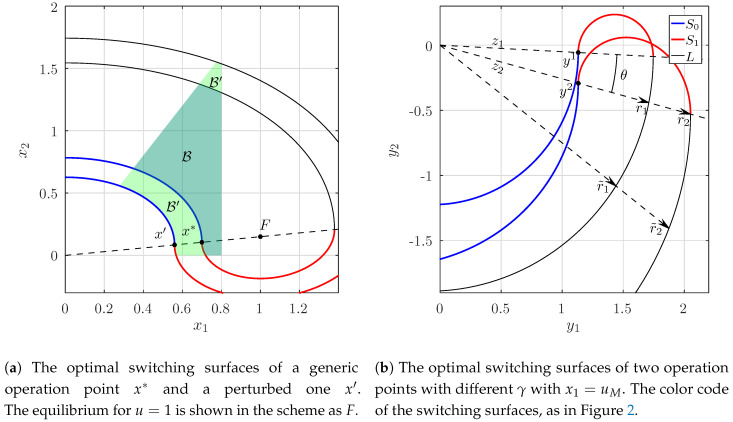
Scheme of the problem statement.

As stated before, the switching surface $S_{1}$ is also a trajectory with a focus in *F*. Since the equilibrium point $x^{*}$ lays along the line $\bar{OF}$ it can be expressed as $x^{*}=cF,\;0\leq c\leq 1$ (see Figure 3a). Now, let us define the new operation point $x^{\prime }:=c^{\prime }F$ with $c^{\prime }<c$, with polar coordinates $r_{0}^{\prime },\alpha_{0}^{\prime }$ and $\alpha_{0}^{\prime }=\alpha_{0}$. With these considerations the following quotient is always guaranteed to be larger than 1:

(18) $$\begin{array}{c} \frac{r_{0}^{\prime }}{r_{0}}=\frac{∥P^{-1}\left(c^{\prime }F-F\right)∥}{∥P^{-1}\left(cF-F\right)∥}=\frac{1-c^{\prime }}{1-c}>1. \end{array}$$

This implies that the switching surface $S_{1}^{x^{*}}$, which corresponds to the optimal surface for $x^{*}$, is always at the interior (respect to the focus *F*) of the switching surface $S_{1}^{x^{\prime }}$. Let us build the sets $B^{\prime }$ and $C^{\prime }$, which correspond to the operation point $x^{\prime }$ in the same manner as B,C were built for $x^{*}$ (see Figure 3a). From (18) we deduce that $B\subset B^{\prime }$ and, consequently, $C⊃C^{\prime }$.

Using this fact, and recalling Equation (14) we obtain:

(19) $$\begin{array}{c} C_{0}=\underset{x\in \{P_{g}|x_{1}=u_{M}\}}{⋃}C_{x}. \end{array}$$

This means that we are able to construct the set $C_{0}$ only by making use of the operation points laying on the line $x_{1}=u_{M}$.

Next, we show that $C_{0}$ can be calculated by making use of a single operation point on the line $x_{1}=u_{M}$, corresponding to the smallest value of the load, namely $\gamma_{1}$. To do this, we need to demonstrate that given two points $x^{1}=\left(x_{1}^{1},x_{2}^{1}\right),x^{2}=\left(x_{1}^{2},x_{2}^{2}\right)$ of $P_{g}$, it turns out that

(20) $$\begin{array}{c} \forall x^{1}\forall x^{2}\in P_{g}:\;x_{1}^{1}=x_{1}^{2},\;x_{2}^{1}<x_{2}^{2}\Rightarrow C_{x^{1}}⊃C_{x^{2}}, \end{array}$$

Let $x^{1}$ and $x^{2}$ be chosen as from Equation (20). Let us pass to the canonically transformed system via the matrix $P_{x^{1}}^{-1}$ according to Equation (15). We now prove that, for every ray from *O*, the radial distance ${\tilde{r}}_{1}$ to the point of the trajectory *L* of $y^{1}$ is always smaller than ${\tilde{r}}_{2}$—that of $y^{2}$ (see Figure 3b). Since $\gamma_{2}>\gamma_{1}$, and so $\frac{\lambda_{2}}{\gamma_{2}}<\frac{\lambda_{1}}{\gamma_{1}}$, we can estimate the quotient between those two distances, as follows:

$$\frac{{\tilde{r}}_{1}}{{\tilde{r}}_{2}}=\frac{r_{1}e^{-\left(\alpha +\alpha_{0}\right)\frac{\lambda_{1}}{\gamma_{1}}}}{r_{2}e^{-\left(\alpha +\alpha_{0}\right)\frac{\lambda_{2}}{\gamma_{2}}}}<\frac{r_{1}}{r_{2}},$$

where $r_{1},r_{2}$ are the initial radii and $\alpha_{0}$ the angle, see Figure 3b. Accordingly, it is sufficient to show $\frac{r_{1}}{r_{2}}<1$.

Secondly, we define the vector $v_{2}={\left(1,\;\gamma_{2}\right)}^{T}$, which points to the focus that generates the switching surface $S_{1}^{x^{2}}$. Under the canonical transformation $v_{2}$ is mapped to $z_{2}=P_{x^{1}}^{-1}v_{2}$. Therefore, the radial distance $r_{2}$ to the endpoint of $S_{1}^{x^{2}}$ can be expressed as

(21) $$\begin{array}{cc} r_{2} & =∥z_{2}∥\left(1-u_{M}\right)e^{-\pi \frac{\lambda_{2}}{\mu_{2}}}+∥z_{2}∥=∥z_{2}∥\left(1+\left(1-u_{M}\right)e^{-\pi \frac{\lambda_{2}}{\mu_{2}}}\right). \end{array}$$

Here, we used the fact that the vectors to the endpoints of the surfaces $S_{1}^{x^{1,2}}$ and corresponding vectors $v_{1,2}$ are colinear. Similarly, for the vector $v_{1}={\left(1,\;\gamma_{1}\right)}^{T}$, which points to the focus generating the switching surface $S_{1}^{x^{1}}$, is mapped to $z_{1}=P_{x^{1}}^{-1}v_{1}$ and the radial distance $r_{1}$ is:

(22) $$\begin{array}{cc} r_{1} & =\left(∥z_{1}∥\left(1-u_{M}\right)e^{-\pi \frac{\lambda_{1}}{\mu_{1}}}+∥z_{1}∥\right)e^{-\theta \frac{\lambda_{1}}{\mu_{1}}}=∥z_{1}∥\left(1+\left(1-u_{M}\right)e^{-\pi \frac{\lambda_{1}}{\mu_{1}}}\right)e^{-\theta \frac{\lambda_{1}}{\mu_{1}}}, \end{array}$$

where θ is the angle between $z_{1}$ and $z_{2}$:

(23) $$\begin{array}{c} \theta =\arccos\frac{\langle z_{1},z_{2}\rangle }{∥z_{1}∥∥z_{2}∥}, \end{array}$$

and

(24) $$\begin{array}{c} ∥z_{1}∥=\frac{2}{\sqrt{4-\gamma_{1}^{2}}}\;\mathrm{and}\;∥z_{2}∥=\frac{2\sqrt{\gamma_{2}^{2}-\gamma_{1}\gamma_{2}+1}}{\sqrt{4-\gamma_{1}^{2}}}. \end{array}$$

Therefore, we have:

(25) $$\begin{array}{c} \frac{r_{1}}{r_{2}}\leq \frac{∥z_{1}∥}{∥z_{2}∥}e^{-\theta \frac{\lambda_{1}}{\mu_{1}}}=\frac{e^{-\theta \frac{\lambda_{1}}{\mu_{1}}}}{\sqrt{\gamma_{2}^{2}-\gamma_{1}\gamma_{2}+1}}. \end{array}$$

After the application of the mean value theorem and some straightforward algebra it can be seen that for every $\gamma_{2}>\gamma_{1}$, the r.h.s of Equation (25) leads to $\frac{r_{1}}{r_{2}}<1$. From this inequality, it is concluded that the set C constructed with the operation point with the smallest possible γ contains all of the inadmissible regions of operation points with larger γ:

(26) $$x_{1}^{1}=x_{1}^{2}=u_{M},\;x_{2}^{1}<x_{2}^{2}\Rightarrow C_{x^{2}}\subset C_{x^{1}}.$$

Hence, the set $C_{0}$ (see Equation (19)) is built using the optimal trajectory passing through $x=(u_{M},u_{M}\gamma_{1})$:

(27) $$C_{0}=\underset{x=(u_{M},u_{M}\gamma_{1})}{C_{x}}$$

**Figure 4:**
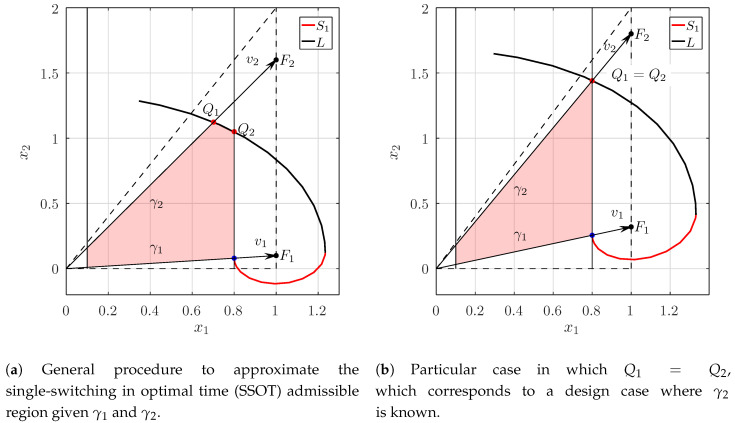
$P_{p}$ regions in two sample design cases.

Now, we are left with approximating the admissible region $P_{p}$ which can be made calculating the intersection of *L* with the boundaries of the region $P_{g}$, which we denote with the points $Q_{1}$ and $Q_{2}$ (see Figure 4a). Let us start with $Q_{1}$ the intersection between *L* and the segment $OF_{2}$ with $F_{2}=\left(1,\gamma_{2}\right)$. One can verify that the coordinate $Q_{1}$ can be expressed as:

(28) $$\begin{array}{c} Q_{1}=cF_{2},\;\mathrm{with}\;c=\frac{|OQ_{1}|}{|OF_{2}|}=\frac{1}{\sqrt{\gamma_{2}^{2}-\gamma_{1}\gamma_{2}+1}}\left(1+\left(1-u_{M}\right)e^{\frac{\pi \gamma_{1}}{\sqrt{4-\gamma_{1}^{2}}}}\right)e^{\frac{\theta \gamma_{1}}{\sqrt{4-\gamma_{1}^{2}}}}. \end{array}$$

Similarly, $Q_{2}$ can be obtained by solving the angle θ, when the $x_{1}$ coordinate of the point in the trajectory *L* reaches $u_{M}$. This can be found by solving the following equation with respect to θ:

(29) $$\begin{array}{cc} \langle r_{1}P\left(\begin{array}{c} \cos(\alpha_{0}-\theta )\\ \sin(\alpha_{0}-\theta ) \end{array}\right),\left(\begin{array}{c} 1\\ 0 \end{array}\right)\rangle & =u_{M}, \end{array}$$

(30) $$\begin{array}{cc} r_{1}\langle \left(\begin{array}{c} \cos(\alpha_{0}-\theta )\\ \sin(\alpha_{0}-\theta ) \end{array}\right),P^{T}\left(\begin{array}{c} 1\\ 0 \end{array}\right)\rangle & =u_{M}. \end{array}$$

where $\alpha_{0}=\arg\left(P_{x^{1}}^{-1}v_{1}\right)=\arctan\frac{2-\gamma_{1}^{2}}{\gamma_{1}\sqrt{4-\gamma_{1}^{2}}}$. Substituting the expressions for $r_{1},\lambda_{1},\mu_{1}$ and *P* we obtain:

(31) $$\frac{2}{\sqrt{4-\gamma_{1}^{2}}}\left(1+\left(1-u_{M}\right)e^{\frac{\pi \gamma_{1}}{\sqrt{4-\gamma_{1}^{2}}}}\right)e^{\frac{\theta \gamma_{1}}{\sqrt{4-\gamma_{1}^{2}}}}\left(\frac{\gamma_{1}}{2}\cos\left(\alpha_{0}-\theta \right)+\frac{\sqrt{4-\gamma_{1}^{2}}}{2}\sin\left(\alpha_{0}-\theta \right)\right)=u_{M}.$$

Let the solution of θ in Equation (31) be $\bar{\theta }$, this allows for us to express the coordinates of $Q_{2}$ as

(32) $$Q_{2}=\left(u_{M},\frac{u_{M}\cos\left(\alpha_{0}-\bar{\theta }\right)}{\sin\left(\arcsin\frac{\gamma_{1}}{2}+\alpha_{0}-\bar{\theta }\right)}\right).$$

Once $Q_{1}$ and $Q_{2}$ have been obtained, a linear fit can be performed between these two points to straightforwardly approximate the curved segment of the trajectory. The resulting $P_{p}$ region after this procedure is shown in the red shaded area in Figure 4a.

## 4. Examples with Predefined Design Criteria

Now, we make use of the analytic approximation to the SSOT region $P_{p}$ to describe some applications in the design of a buck converter. Very often, a power converter is designed with nominal specifications of maximum or minimum working load. A useful question that the geometrical methodology that we proposed here can answer is: given that there is a predefined maximum (or minimum) load, what is the smallest (largest) variation of the load such that the system is still able to reach the operation point in SSOT?

Let us first focus on the case in which a load increase is presented to the system. If the converter is working at minimum nominal load, which we denote as $\gamma_{1}$, what is the $P_{p}$ region if the system is allowed to vary its load to the maximum allowed $\gamma_{2}$? Here, both $\gamma_{1}$ and $\gamma_{2}$ are given, and we are only left with calculating the points $Q_{1}$ and $Q_{2}$ as explained in Section 3. For instance, introducing the values of $\gamma_{1}=0.1$ and $\gamma_{2}=1.6$ in Equations (28) and (32), leads to the coordinates $Q_{1}=\left(0.5936,1.1872\right)$ and $Q_{2}=\left(0.8,1.05\right)$ which is precisely the case that is depicted in Figure 4a.

Similarly, the second problem refers to the case in which the converter is working with the maximum nominal load, which we will set to $\gamma_{2}$ and we want to calculate the minimum allowed change in the load $\gamma_{1}$ such that the region bounded by $\gamma_{1}$ and $\gamma_{2}$ leads to the SSOT region. Here, $\gamma_{2}$ is known and $\gamma_{1}$ is unknown. However from Figure 4b, it can be seen that set-up corresponds to the case in which $Q_{1}=Q_{2}$. For this case we only need to set $c=u_{M}$ in Equation (28) and solve with respect to $\gamma_{1}$:

(33) $$\begin{array}{c} \frac{u_{M}\sqrt{\gamma_{2}^{2}-\gamma_{1}\gamma_{2}+1}}{\left(1+\left(1-u_{M}\right)e^{\frac{\pi \gamma_{1}}{\sqrt{4-\gamma_{1}^{2}}}}\right)}=e^{\frac{\theta \gamma_{1}}{\sqrt{4-\gamma_{1}^{2}}}}. \end{array}$$

**Figure 5:**
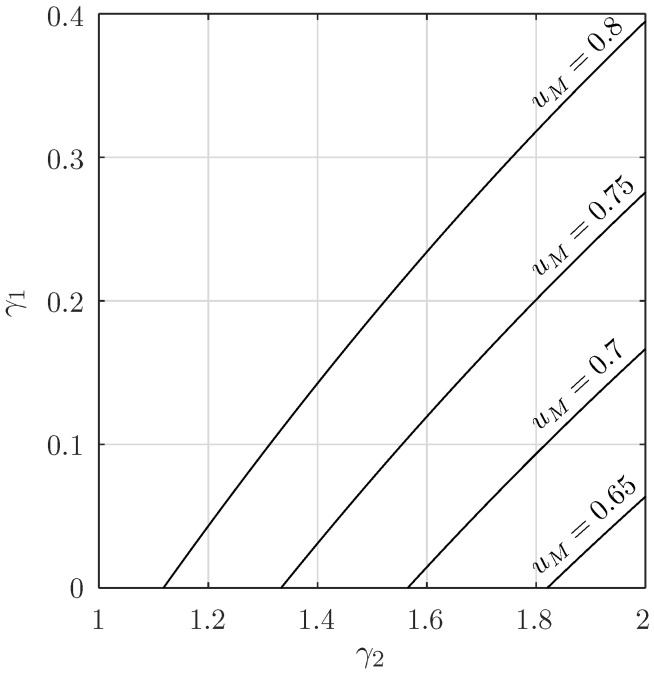
$\gamma_{1}$ calculated with respect to a given $\gamma_{2}$ in the special case $Q_{1}=Q_{2}$ (see Figure 4b).

For $\gamma_{2}=1.8$ and $u_{M}=0.8$, this results in $\gamma_{1}\approx 0.32$, as can depicted in Figure 4b. Of course, this last calculation depends on the maximum output voltage $u_{M}$. In Figure 5, we can see the variation of $\gamma_{1}$ with respect to $\gamma_{2}$ at different $u_{M}$. Note, that for $u_{M}\approx 0.618$, $\gamma_{2}=2$ results in $\gamma_{1}=0$, in other words, the corresponding region $C_{0}$ (the inadmissible region of $P_{g}$) is indeed empty. An alternative derivation of the $P_{p}$ region of these two sample design criteria using the original formulation of the system without canonical transformations can be found in the Appendix A.

## 5. Numerical Simulations

In this section, we show the numerical results for two different working conditions, namely under variations of the reference voltage and the system’s load. The parameters that are used for the simulation are: L=2 mH, C=40μF, and E=40 V. Variations in γ are obtained varying *R*. The switching period of the MOSFET is assumed to be $T_{s}$ = 25 μs, which is equivalent to T=0.08835 in the dimensionless system according to the transformation $t=\tau /\sqrt{LC}$.

### 5.1. Changes in the Reference Voltage and Load

**Figure 6:**
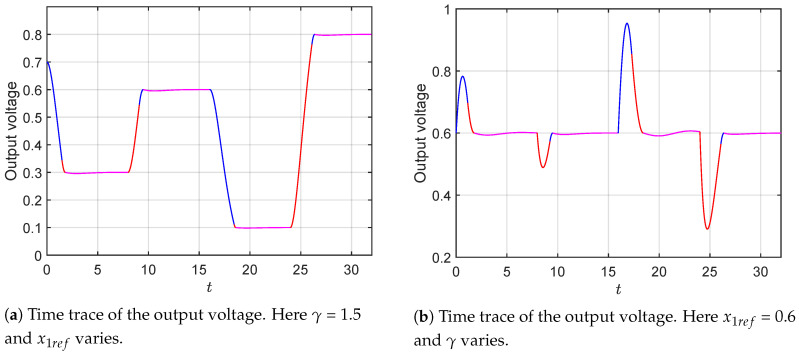
Independent variations in the reference voltage and in the load parameter. In both figure the changes are performed every eight time units. The blue (red) segment of the trace corresponds to the dynamics of the converter with u=0 (u=1). The purple segment illustrates the dynamics in the neighborhood of the operation point driven by a high frequency proportional controller. In (**a**), $x_{1ref}$ changes from $x_{1ref}=0.7$ to $x_{1ref}=0.3$ to $x_{1ref}=0.6$ to $x_{1ref}=0.1$ to $x_{1ref}=0.8$ keeping a value of γ=1.5. In (**b**), γ changes from γ=1.75 to γ=0.75 to γ=1.5 to γ=0.2 to γ=1.88, while keeping $x_{1ref}=0.6$.

On one hand, in Figure 6a, we show the results obtained by applying the time optimal control to the system, assuming a constant load (constant γ) with several sudden variations in the reference voltage. As can be seen from the figure, the output of the system reaches the final value in optimal time (for the cases considered here this time corresponds roughly to three to four dimensionless time units). Moreover, it is also possible to observe that a single switch is used to control the system (see transition from blue to red in the time trace). Once the system has reached the desired value, a high frequency controller needs to be used to maintain the output close to the reference point (purple region). For this particular case, we have made use of proportional controller to compensate the duty cycle at the reference value and supply the value of the duty cycle in steady state. On the other hand, in Figure 6b, we show the results when the reference voltage is held constant ($x_{1ref}=0.6$) and γ varies. As in the previous case, the system converges to the operation point in optimal time which is roughly the same, as in the case of reference variations. It can be observed that the system handles better the changes in the reference when compared with the variations in the load (see larger over/under-shoots) in Figure 6b with respect to Figure 6a. This observation can be understood on the basis that a change in the load implies a change in the intrinsic dynamics of the system, while changes solely in the reference does not. It shall be noticed that the small oscillations observed in the purple traces are a consequence of the finite commutation frequency of the MOSFET. An ideal switch with infinite frequency would lead to a completely flat segment at the reference value.

**Figure 7:**
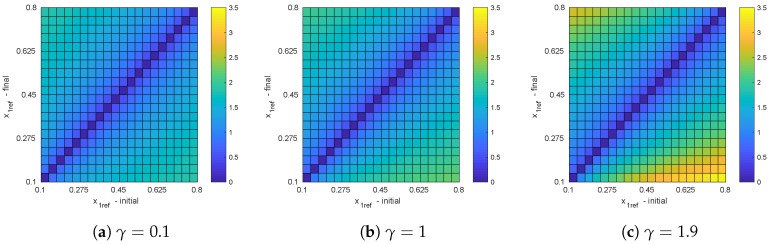
Settling times for different variations of $x_{1ref}$ at fixed values of γ.

With the aim of determining the time taken by the system to reach the steady operation at different $x_{1ref}$ variations, we show in Figure 7 the settling times when the system transits from an initial reference value to another, at several values of fixed load, namely, γ=0.1, γ=1, and γ=1.9. For a given γ, the maximum settling time is obtained for step-down transitions, i.e., for changes from a large reference to a lower value. It can be also noticed that in general the settling time tends to increase for increasing values of γ, leading to the largest value when the load resistance is minimal. This is not surprising, since the natural frequency of oscillation of the system is related with the quantity $\mu =\frac{\sqrt{4-\gamma^{2}}}{2}$ (see Equation (16)), which increases with decreasing γ. Currently, since the settling time of the system closely depends on how fast the trajectory evolves towards the optimal switching surface, it is expected that systems with faster intrinsic dynamics lead to smaller settling times.

**Figure 8:**
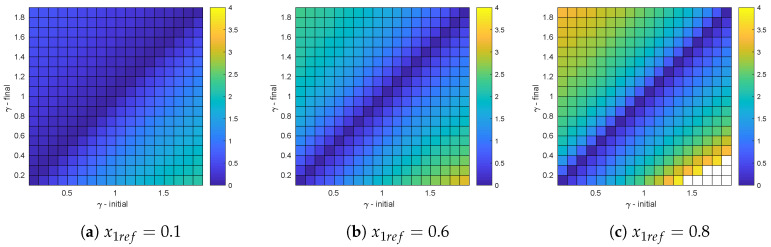
Settling times for different variations of γ at fixed values of $x_{1ref}$.

Similarly, Figure 8 depicts the settling times between different transitions of γ when $x_{1ref}$ is fixed. In this case as $x_{1ref}$ increases the settling time does. This behavior is explained by the fact that the distance between the operation points when transitioning from one γ to another, are small when compared with the same transition at large $x_{1ref}$ values. From the trend of these graphics, it is expected that the worst expected settling times required to move from an operation point to another within the admissible region $P_{p}$, corresponds to the transition from upper right corner operation points to the lower left ones. It shall be noticed that the uncolored region in Figure 8c correspond to transitions outside $P_{p}$ that require more than one switching action.

### 5.2. Simultaneous Changes in the Reference Voltage and Load

**Figure 9:**
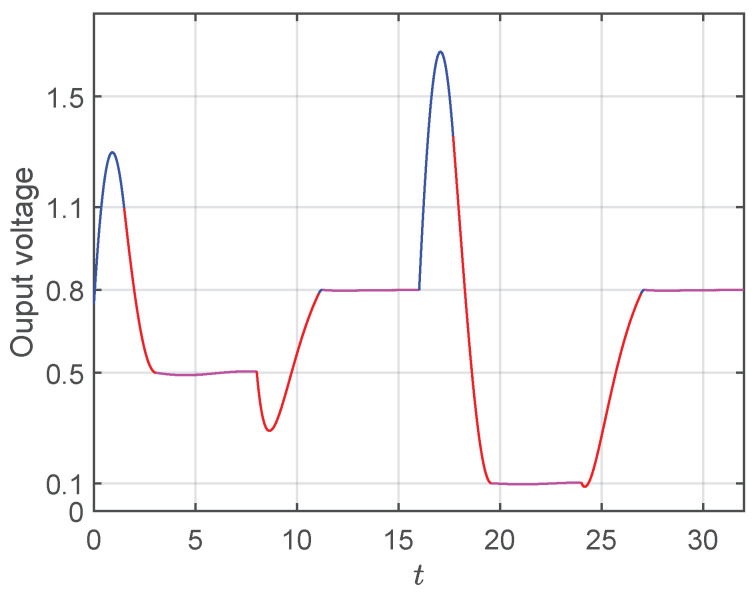
Time traces of the output voltage for simultaneous variations of γ and $x_{1ref}$. Here, four sudden changes are shown at t=0, t=8, t=16 and t=24 characterized by the following variations of the duple $\left(x_{1ref},\gamma \right)$: (0.75,1.9)→(0.5,0.32)→(0.8,1.99)→(0.1,0.1)→(0.8,1.99). The color code of the time trace, as in Figure 6.

Finally, we are interested in analyzing the behavior of the system when both parameters, load resistance, and reference voltage change simultaneously. In particular, in Figure 9 we simulate the system with the following variations: At t=0 the system starts from $x_{1ref}=0.75$, γ=1.9 and the new desired operation point is changed to $x_{1ref}=0.5$ and γ=0.32. After, at t=8, $x_{1ref}$ changes to 0.8 and γ to 1.99. Just at t=16 other change is introduced and $x_{1ref}=0.1$ and γ=0.1. Finally, at t=24, a further change is performed with $x_{1ref}=0.8$, γ=1.99. As in the previous subsection, a high frequency proportional controller has been applied when the the trajectory reaches the neighborhood of the operation point. Once again, the settling times are ≈3 time units, which correspond in real units to settling times in the order of 1 ms.

## 6. Conclusions and Final Remarks

In this paper we made use of the Pontryagin’s maximum principle to derive the admissible region $P_{p}$ in the state space of a synchronous buck DC/DC converter, which can be reached in optimal time with a single commutation (SSOT) from any point within the same region. In doing so, we made use of a geometric analysis of the trajectories described by the buck converter in canonical coordinates to better understand the admissible subset of the phase plane fulfilling the SSOT condition. Furthermore, we studied two different design set-ups of the buck converter, and used the results obtained previously in order to illustrate the usage of our calculations. Finally, we showed, by means of numerical simulations, that the proposed time optimal control performs adequately under voltage and load variations, both independently and simultaneous.

The most critical procedure in the design of the optimal control studied here is the ability to calculate on-line the proper commutation time, i.e., the time in which the trajectory hits the optimal switching surface, which strongly depends on parameter variations, as explained throughout the text. This problem has been previously identified and several solutions proposed in the form of look-up tables [11], near-time optimal solutions [25] and raster surfaces [26]. These methodologies can be used as a complement to the theoretical derivations presented here.

The difficulty of calculating the switching times online is nonetheless compensated with a significant reduction in the settling times of the system. In the simulations shown here, we have shown settling times *t*∼3 time units (less than 1 ms in real time units). As a comparison, the same buck converter model with similar parameter values has been recently used to design a globally stable controller [10]. The settling times reported there are 10 times larger than those shown here. It shall be noticed that γ contains the information of the system’s parameters in a single value. This amounts to say that different combinations of *L* and *C* can be chosen in such a way to modify the time scale of the system’s response and the ranges of *R* in which the buck converter can operate under the damped conditions studied here.

It is also worth noticing that the optimal control presented here is useful during the transient behavior following a perturbation from the usual operation point. After the trajectory has reached the neighborhood of this point, we are constrained to use high frequency regulation in order to maintain the trajectory within the neighborhood. Indeed, we saw that the system’s performance in steady state strongly depends on the commutation frequency of the MOSFET. Limitations of the commutation frequency produces undesired oscillations around the operation point and effectively increases the settling times. Additional controllers, such as P or PIs in the high frequency steady operation, can be used in order to better handle undesired oscillations and/or reject disturbances. Additionally, further optimization can be performed during this high frequency regime, which we expect to explore in the future.

Throughout this manuscript, we have chosen to study the buck converter as a proof-of-concept to extend the ideas of estimating the SSOT regions in other physical problems of interest. We predict that such an estimation can be also performed in other piece-wise linear converters, such as boost, buck-boost, and boost-flyback converters by making use of a similar geometrical approach shown here. However, it shall be noticed that most power converters (boost, buck-boost, and flyback) are different from the buck converter from a mathematical perspective. For instance in the buck converter the action of the switching action amounts to shift the equilibrium without changing its properties. Meanwhile, the commuting action in the other converters produce changes in the topology of the system (different eigenvalues), a property that may require additional calculations.

## Appendix A

In this appendix we reproduce the results in Section 4 making use of the original system and using a more intuitive numerical approach (although it must be noticed that the methods are equivalent). Let us start rewriting the original system here:

(A1) $$\left(\begin{array}{c} \dot{v}\\ \dot{i} \end{array}\right)=\left(\begin{array}{cc} -\frac{1}{RC} & \frac{1}{C}\\ -\frac{1}{L} & 0 \end{array}\right)\left(\begin{array}{c} v\\ i \end{array}\right)+\left(\begin{array}{c} 0\\ \frac{E}{L} \end{array}\right)u$$

With L=2 mH, C=40μF and *E* = 40 V. *R* varies according to the changes in the load and its limits are given by the limits of γ value in the problem statement. Different choices of *L* and *C* change also the range of variation of *R*. To compute the solution of the proposed problem using these equations, we recall that:

1. $\gamma =\frac{1}{R}\sqrt{\frac{L}{C}}$. As the changes are given in the load (resistance), we fix the values of *L* and *C* and compute the limiting value of $R_{lim}$ such that it results in a γ=2. This will correspond to the *lowest* admissible value for *R*.
2. As γ is inversely proportional to *R*, $\gamma_{1}$ corresponds to the highest value of the resistance and $\gamma_{2}$ corresponds to the lowest value of the load resistance.
3. $x_{1ref}=v_{ref}/E$; then $\;\;0.1E\;<\;v_{ref}\;<0.8E$. This means that after the value of the source power (*E*) is known, the limits of the $v_{ref}$ are known too.
4. With an adequate switching frequency, any reference point given by $\left(v^{*},\;\;i^{*}\right)=\left(v_{ref},\;\;v_{ref}/R\right)$ can be reached, where $v_{ref}=\bar{u}E$
5. The two equilibria of the system according to the switch position are (E,E/R) when u=1, and (0,0), when u=0.

**Subregion $P_{p}$ for $R\in [R_{\mathrm{low}},\;\;R_{\mathrm{high}}]$, where $R_{\mathrm{low}}$ and $R_{\mathrm{high}}$ are known**. This case corresponds to the case in which $\gamma_{1}$ and $\gamma_{2}$ are known. We propose to find the set of reachable points starting from $0.8E\;(1,\;1/R_{high})$. To solve the problem it is necessary to find two key points: The first one is given by the coordinates of the initial condition $v_{ref_{IC}}{\left(1,\;\;1/R_{low}\right)}^{T}$ with $v_{ref_{IC}}$ unknown, whose orbit ends at the point $0.8E\;{\left(1,\;\;1/R_{high}\right)}^{T}$ when $R=R_{high}$ after $\tau =\tau_{1}+T/2$ and the control action takes the sequence u=0→u=1. This is equivalent to the point $Q_{2}$ in Figure 4a. The second point is given by the intersection of this orbit with the line $v_{ref}=0.8E$ (see point $Q_{1}$ in the same figure). The upper part of the region $P_{g}$ is finally bounded -at least approximately- by a straight line from the first point to second one. With this in mind, we proceed to find the first point solving the matrix equation

(A2) $$\begin{array}{ccc} 0.8E\;\left(\begin{array}{c} 1\\ 1/R_{high} \end{array}\right) & = & v_{ref_{IC}}e^{A(T/2+t_{1})}\left(\begin{array}{c} 1\\ 1/R_{low} \end{array}\right)+A^{-1}\left(e^{AT/2}-I\right)B \end{array}$$

with $T/2=2\pi R_{high}C\sqrt{L}/\sqrt{4R_{low}^{2}C-L}$. After, the second point is given by the intersection of the solution starting from $v_{ref_{IC}}{\left(1,\;\;1/R_{low}\right)}^{T}$ with the line given by v=0.8E after an unknown time $\tau_{2}$. This point has coordinates 0.8E(1,1/r), where *r* is unknown. To solve this we need to find the solution of the equation

(A3) $$\begin{array}{ccc} 0.8E\;\left(\begin{array}{c} 1\\ 1/r \end{array}\right) & = & v_{ref_{IC}}\;e^{At_{2}}\left(\begin{array}{c} 1\\ R_{low} \end{array}\right) \end{array}$$

Once we have solved for *r* and $\tau_{2}$ we can give and approximated value to the region $P_{p}$ as

(A4) $$\begin{array}{ccc} P_{p} & = & \left\{i\geq v/R_{high}\right\}\;\cap \;\left\{0.1E\leq v\leq 0.8E\right\}\;\cap \;\left\{i\leq v/R_{low}\right\}\;\cap \;\left\{i\leq mv+b\right\} \end{array}$$

where $b=v_{ref_{IC}}\left(1-m\;R_{low}\right)/R_{low}$ and

$$m=\frac{0.8E\;R_{low}-v_{ref_{IC}}\;r}{\left(0.8E-v_{ref_{IC}}\right)\left(R_{low}\;r\right)}.$$

**Subregion $P_{p}$ for $R\in [R_{\mathrm{low}},\;\;R_{\mathrm{high}}]$ with $R_{\mathrm{low}}$ known**. This corresponds to the case in which $\gamma_{2}$ ($R_{low}$) is known and $R_{high}$ ($\gamma_{1}$) is unknown. To compute $R_{high}$ we take the point $0.8E\;{\left(1,\;\;1/R_{low}\right)}^{T}$ which corresponds to upper point on the region $P_{g}$, which lies in the intersection between the lines v=0.8E, and $i=v/R_{low}$. With this condition and making u=0, the system evolves during an unknown time $\tau_{1}$ to reach the unknown point $v\left(\tau_{1}\right){\left(1,\;\;1/R_{high}\right)}^{T}$. The solution can be easily found as:

(A5) $$X:=v\left(\tau_{1}\right)\left(\begin{array}{c} 1\\ 1/R_{high} \end{array}\right)=0.8Ee^{A\tau_{1}}\left(\begin{array}{c} 1\\ 1/R_{low} \end{array}\right)$$

Here $e^{A\tau }$ is the state transition matrix of the system when $R=R_{high}$. Now, starting from this point and considering u=1 during a time $\tau =T/2=2\pi R_{high}C\sqrt{L}/\sqrt{4R_{high}^{2}C-L}$ (corresponding to the oscillation period time for the damped system), the solution must reach the desired final point $0.8E\;{\left(1,\;\;1/R_{high}\right)}^{T}$; that is, the following equation must be satisfied:

(A6) $$0.8E\left(\begin{array}{c} 1\\ 1/R_{high} \end{array}\right)=e^{AT/2}X+A^{-1}\left(e^{AT/2}-I\right)B$$

where *A* and *B* are defined by the Equation (1) and *I* is the identity matrix of dimension 2. The previous equation can be expressed as

(A7) $$\begin{array}{ccc} 0.8E\left(\begin{array}{c} 1\\ 1/R_{high} \end{array}\right) & = & 0.8E\;e^{A(T/2+\tau_{1})}\left(\begin{array}{c} 1\\ R_{low} \end{array}\right)+A^{-1}\left(e^{AT/2}-I\right)B \end{array}$$

where we are left with the unknown variables are $R_{high}$ and $t_{1}$. In this way, the region $P_{p}$ is given by:

(A8) $$\begin{array}{ccc} P_{p} & = & \left\{i\;\geq \;x_{1}/R_{high}\right\}\;\cap \;\left\{0.1\;E\leq v\leq \;0.8\;E\right\}\;\cap \;\left\{i\;\leq \;x_{1}/R_{low}\right\} \end{array}$$

similar to the Figure 4b.
